# Monitoring Matters: Physical Health Monitoring for Children and Young People Prescribed Antipsychotics in Community CAMHS

**DOI:** 10.1192/bjo.2026.11621

**Published:** 2026-06-30

**Authors:** Gabriella Marlow

**Affiliations:** Sheffield Health Partnership University NHS Trust, Sheffield, United Kingdom. Sheffield Children's Hospital NHS Trust, Sheffield, United Kingdom

## Abstract

**Aims:**

This audit aimed to evaluate adherence to the Community Child and Adolescent Mental Health Services (CAMHS) Physical Health Standard Operating Procedure for monitoring patients prescribed antipsychotic medication within Centenary House CAMHS. The hypothesis was that recommended physical health monitoring would be incomplete for a substantial proportion of patients.

**Methods:**

A retrospective service evaluation was conducted of all patients under medical care at Centenary House CAMHS who were prescribed antipsychotic medication during the audit period (n=13).

Electronic clinical records were reviewed using structured searches within the SystmOne electronic health record and ICE blood results system. Evidence of baseline physical health monitoring was sought; where baseline data were unavailable (for example, due to out-of-area initiation), monitoring within the preceding six months was assessed.

The parameters audited were appropriate blood tests for antipsychotic monitoring, electrocardiogram (ECG), height and weight, blood pressure and pulse, documented neurological examination for EPSEs, and allergy status. No patients meeting inclusion criteria were excluded.

This work was undertaken as a service evaluation audit with appropriate local approval and anonymisation of patient data.

**Results:**

Thirteen patients were identified; ten prescribed aripiprazole, two quetiapine and one risperidone. Documented compliance rates were 77% (10/13) for blood tests, 85% (11/13) for electrocardiogram, 62% (8/13) for height and weight, 62% (8/13) for blood pressure and heart rate, and 62% (8/13) for allergy status. No patient had a documented neurological examination for EPSEs. Only six patients had all required physical health parameters documented either at baseline or within the previous six months. Prolactin monitoring was absent in the single patient prescribed risperidone.

**Conclusion:**

Physical health monitoring for children and young people prescribed antipsychotics within this community CAMHS was variable and frequently incomplete, particularly for neurological assessment and consistent documentation. Service improvements are required to standardise monitoring and improve patient safety.

Proposed next steps include

1. Implementation of electronic reminders

2. Introduction of a structured antipsychotic monitoring template

3. Targeted teaching for medical staff and

4. Planned re-audit within six to twelve months to assess improvement.

